# In-vitro propagation and phytochemical profiling of a highly medicinal and endemic plant species of the Himalayan region (*Saussurea costus*)

**DOI:** 10.1038/s41598-021-03032-1

**Published:** 2021-12-08

**Authors:** Ajmal Khan, Azhar Hussain Shah, Niaz Ali

**Affiliations:** 1 Department of Botany, Hazara University Mansehra, Mansehra, KP Pakistan; 2 Department of Biotechnology and Genetic Engineering, Hazara University Mansehra, Mansehra, KP Pakistan

**Keywords:** Tissue engineering, Plant regeneration

## Abstract

Efficient protocols for callus induction and micro propagation of *Saussurea costus* (Falc.) Lipsch were developed and phytochemical diversity of wild and in-vitro propagated material was investigated. Brown and red compact callus was formed with frequency of 80–95%, 78–90%, 70–95% and 65–80% from seeds, leaf, petiole and root explants, respectively. MS media supplemented with BAP (2.0 mgL^−1^), NAA (1.0 mgL^−1^) and GA3 (0.25 mgL^−1^) best suited for multiple shoot buds initiation (82%), while maximum shoot length was formed on media with BAP (1.5 mgL^−1^), NAA (0.25 mgL^−1^) and Kinetin (0.5 mgL^−1^). Full strength media with IAA (0.5 mgL^−1^) along with IBA (0.5 mgL^−1^) resulted in early roots initiation. Similarly, maximum rooting (87.57%) and lateral roots formation (up to 6.76) was recorded on full strength media supplemented with BAP (0.5 mgL^−1^), IAA (0.5 mgL^−1^) and IBA (0.5 mgL^−1^). Survival rate of acclimatized plantlets in autoclaved garden soil, farmyard soil, and sand (2:1:1) was 87%. Phytochemical analysis revealed variations in biochemical contents i.e. maximum sugar (808.32 µM/ml), proline (48.14 mg/g), ascorbic acid (373.801 mM/g) and phenolic compounds (642.72 mgL^−1^) were recorded from callus cultured on different stress media. Nonetheless, highest flavenoids (59.892 mg/g) and anthocyanin contents (32.39 mg/kg) were observed in in-vitro propagated plants. GC–MS analysis of the callus ethyl acetate extracts revealed 24 different phytochemicals. The variability in secondary metabolites of both wild and propagated plants/callus is reported for the first time for this species. This study may provide a baseline for the conservation and sustainable utilization of *S. costus* with implications for isolation of unique and pharmacologically active compounds from callus or regenerated plantlets.

## Introduction

Plants have been essential sources of medicine for thousands of years and nearly 80% of the world’s population still relies on traditional medicine for their primary healthcare^1^. *Saussurea costus* is an endemic species in geographically limited places of the Himalayas, where it grows on moist slopes at altitudes of 2500–4000 m. The species is critically endangered and is listed in Appendix I of the Convention on International Trade in Endangered Species of Wild Fauna and Flora (CITES). In addition, it is one of the 37 endangered and highly medicinal plants of the Himalayas, and has been prioritized for both *in-situ* and *ex-situ* conservation^2^. *S. costus* is a highly prized medicinal plant in the Kaghan valley Pakistan. Roots of *S. costus* have sweet and strong aromatic odor with bitter taste and are used as antiseptics as well as for treating bronchial asthma, especially of the vagotonic type. The roots of *S. costus* have been widely used for curing diarrhea, jaundice, stomachache, respiratory tract infections, antispasmodic agents against spasms caused by asthma, cholera, rheumatism, chronic skin diseases and leprosy^3^. Further, oil extracted from the roots (referred to as Costus oil) is used for making high grade perfumes and hair oils^4^. In addition, many studies have shown that extracts of *S. costus* have potent anti-cancer, anti-inflammatory and anti-ulcer properties^5^. Because of the high demands for roots, most natural populations of *S. costus* are on the verge of extinction^6^.

In order to avoid the future loss of endangered, endemic and rare species, conservation of plant genetic resources has long been realized as an integral part of biodiversity conservation. Plant cell and tissue culture has been a powerful tool for rapid propagation and biomass production of valuable species. To overcome environmental constraints in-vitro cultures (cell, callus, buds and shoot) provide the best alternative choice for the smooth and constant supply of plant active ingredients^7^. However, there are no effort in literature for *ex-situ* conservation and micro propagation of *S. costus*. Further, phytochemical composition of the wild (natural) *S. costus* and tissue culture generated material is totally non-existent. The purpose of this research was to establish an effective and efficient in vitro regeneration protocol for *S. costus* and to compare the photochemical variability in the aqueous extracts of induced callus, in-vitro propagated plants with the wild/natural collections.

## Materials and Methods

### Plant material and sterilization procedure

Mother plant was collected from wild populations in Makra, Kaghan valley, Pakistan (lat 34.57439º N, long 073.49580º E, alt 3,878 m). The specimen was identified by Dr. Abdul Majid Department of Botany Hazara University Mansehra, and scientific name validated online (http://www.theplantlist.org/). Voucher specimen was submitted to the Herbarium Department of Botany Hazara University, Mansehra. Healthy plant parts (explant) were separated from the mother plant, washed and sterilized following Yesmin et al. (2016)^8^.

### Culture conditions

The basal MS media^9^ was used with various concentration and composition of growth regulators (BAP, IAA, IBA, NAA, 2,4-D and kinetin). All culture media were agitated with 7% technical agar and 3% sucrose. The pH of media was set to 5.8 before addition of agar. These media were autoclaved at 121 °C for 20 min at 15 psi. Cultures were maintained in a culture room incubated with a 16-h light cycle and temperature maintained at 25 ± 2 °C with 50% humidity.

### Callus induction

Growth regulators such as 2,4-D (0.25, 0.5, and 1.0 mgL^−1^) in combination with varied concentration of Kinetin (0.5, 1.0, 1.5 and 2.0 mgL^−1^), and four explants types (seeds, leaf, petiole and internode) were compared for callus induction. Explants were subjected to two subcultures at an interval of fourteen days^10^.

### Shoot bud initiation

Full strength MS media with different concentration of BAP (0.5, l.0, 2.0 mgL^−1^), NAA (0, 0.25, l.0 mgL^−1^) and GA_3_ (0, 0.25, 1.0 mgL^−1^) were compared for shoot buds initiation. The percentage of shoot induction, time taken for bud initiation and the growth state of the buds were measured after four weeks of culturing.

### Shoot proliferation

Nodal segments (1–2 cm long) were excised from cultured plant and transferred into MS media agitated with BAP (0.5, 1.0, 1.5 mgL^−1^) in combination with NAA (0.25, 0.25 and 0.5 mgL^−1^) and Kinetin (0, 0.25 and 0.5 mgL^−1^) in order to maximize shoot multiplication. In addition, basic MS media with different plant growth regulators were compared during the phase of subculture, and the optimal media for shoot proliferation was selected.

### Root initiation

To optimize root induction media, full-strength MS media was supplemented with different combination and concentrations of IAA (0.5, 1.0 mgL^−1^) and IBA (0.5, 0.1 mgL^−1^) along BAP (0.5 mgL^−1^). The time to root initiation was observed and recorded after every two days. Data on average root numbers and length were recorded after 45 days of culturing.

### Photochemical analysis

#### Treatments of in-vitro callus

After subculture for eight cycles, fourteen days old callus was subjected to four different stresses each having five (05) replications. Callus was cultured on callus promoting media (CPM) having 0.5 and 1.0 mgL^−1^ Kinetin and 2.4-D respectively. In addition, callus was cultured on media agitated with 60 gL^−1^ D-Sorbitol stress (CPM-1), 60 gL^−1^ D-Manitol stress (CPM-2), 5 gL^−1^ Poly ethylene glycol 600 stress (CPM-3) and 60 gL^−1^ Sucrose stress (CPM-4) for 120 days, while the callus cultured on CPM alone was considered as control (Table 1). Similarly, different concentrations of growth hormones given to calli for bud, shoot or root induction are given (see Tables 2–5).

**Table 1:** Different stress for phytochemical comparison of callus grown on simple callus promoting media (CPM) supplemented with Kinetin (0.5 mgL^-1^) and 2.4-D (1.0 mgL^-1^) and callus subjected to various stresses i.e. 60 gL^-1^ D-Sorbitol stress (CPM-1), 60 gL^-1^ D-Manitol (CPM-2), 5 gL^-1^ Poly ethylene glycol 600 (CPM-3) and 60 gL^-1^ Sucrose (CPM-4).

Samples	Kinetin (mgL^-1^)	2,4-D (mgL^-1^)	Callus stress
CPM	0.5	1.0	**–-**
CPM-1	0.5	1.0	D-Sorbitol (60 gL^-1^)
CPM-2	0.5	1.0	D-Manitol (60 gL^-1^)
CPM-3	0.5	1.0	Poly ethylene glycol 600 (5 gL^-1^)
CPM-4	0.5	1.0	Sucrose (60 gL^-1^)

**Table 2:** In-vitro callus induction and callus growth after 30 days of culturing of *S. costus* using seed, leaf, petiole and root as an explants.

Treatments	Conc. 2,4-D/Kinetin mgL^-1^	Type of explants used	Means days to callus induction (x- ± SE)	Callus growth after 30 days (x- ± SE)
T1	0.25/0.5	Seeds	14.00 ± 1.09^A^	0.22 ± 0.07^D^
		Leaf	15.20 ± 0.73^C^	1.50 ± 0.08^AB^
		Petiole	15.80 ± 0.73^4^^BC^	0.91 ± 0.02^D^
		Root	16.40 ± 0.97^A^	0.91 ± 0.05B^C^
T2	0.5/0.5	Seeds	17.60 ± 0.67^A^	1.39 ± 0.06^CD^
		Leaf	17.20 ± 0.37^C^	1.34 ± 0.04^AB^
		Petiole	17.80 ± 0.37^AB^	1.27 ± 0.08^BC^
		Root	19.20 ± 0.37^AB^	0.92 ± 0.02^B^
T3	0.5/1.0	Seeds	13.40 ± 0.60^A^	1.86 ± 0.06^A^
		Leaf	15.80 ± 1.07^C^	1.65 ± 0.06^A^
		Petiole	15.60 ± 0.50^C^	1.42 ± 0.05^A^
		Root	17.40 ± 0.86^BC^	1.114 ± 0.07^A^
T4	0.5/1.5	Seeds	16.60 ± 0.60^A^	1.62 ± 0.05^B^
		Leaf	18.60 ± 1.07^AB^	1.32 ± 0.08^AB^
		Petiole	18.60 ± 0.50^A^	1.33 ± 0.02^AB^
		Root	17.20 ± 0.86^BC^	0.75 ± 0.08^C^
T5	0.5/2.0	Seeds	17.40 ± 1.07^A^	1.54 ± 0.08^BC^
		Leaf	18.20 ± 0.66^AB^	1.23 ± 0.06^BC^
		Petiole	18.40 ± 0.50^A^	1.26 ± 0.03^BC^
		Root	19.40 ± 0.74^AB^	0.90 ± 0.01^BC^
T6	1.0/2.0	Seeds	18.20 ± 0.80^A^	1.65 ± 0.06^B^
		Leaf	19.60 ± 0.81^A^	0.92 ± 0.20^C^
		Petiole	19.01 ± 0.89^A^	1.18 ± 0.06^C^
		Root	20.80 ± 0.73^A^	0.84 ± 0.03^BC^

#### Samples preparation

Samples for spectrometry (BMS, UV-1900) were prepared following Storey and Jones (1975)^11^. Total sugars contents were analyzed following Dubois et al. (1956)^12^, proline content was assessed following Bates et al. (1973)^13^, flavenoids were assessed as per Csepregi et al. (2013)^14^. Antioxidant activity was as described in Re et al. (1999)^15^, total phenol contents was measured following Singleton and Rossi (1965)^16^ and total anthocyanin content was determined following Giusti and Wrolstad (2001)^17^.

#### Preparation of solvent extraction for GC–MS

Callus subjected to different stresses (Table 1) as well as grown on CPM was shade dried and grounded to fine powder using mortar and pestle. For solvent preparation 1 g (dry weight) of powder was soaked in 10 ml of ethyl acetate for 2 days. The sample was centrifuged at 8,000 rpm for 5 min and the supernatant collected was stored at 4 °C for further analyses^18^.

#### Gas chromatography-mass spectrometry (GC–MS) analysis

Chemical analysis of ethyl acetate extract was carried out using gas chromatography coupled with mass spectrometry (GC–MS) with a Hewlett Packard GC–MS system (PerkinElmer precisely, Carlus 600C). The relative percentage of each component was calculated by comparing the average GC chromatogram peak to the total area. The mass detector used in this analysis was Turbo-Mass Gold-Perkin-Elmer, and the software adopted to handle mass spectra and chromatograms was a Turbo-Mass ver-5.4^19^.

#### Identification of compounds

Interpretation on mass spectrum GC–MS was conducted using the database of National Institute Standard and Technology (NIST). The spectrum of a component was compared with the spectrum of the known components stored in the NIST library. Similarly, name, molecular weight and structure of the components of the test materials were ascertained^19^.

#### Statistical analysis

Statistical analysis was performed with Statistic 8.1 (Trial version). Results were presented as mean ± standard error (SE), and the data was analyzed by one way Analysis of variance (ANOVA) at 0.05% confidence level (p < 0.01). All in-vitro propagation treatments had 5 replications whereas; the phytochemical analyses had three replications for each treatment.

## Results and discussion

### Callus induction

**Figure 1:**
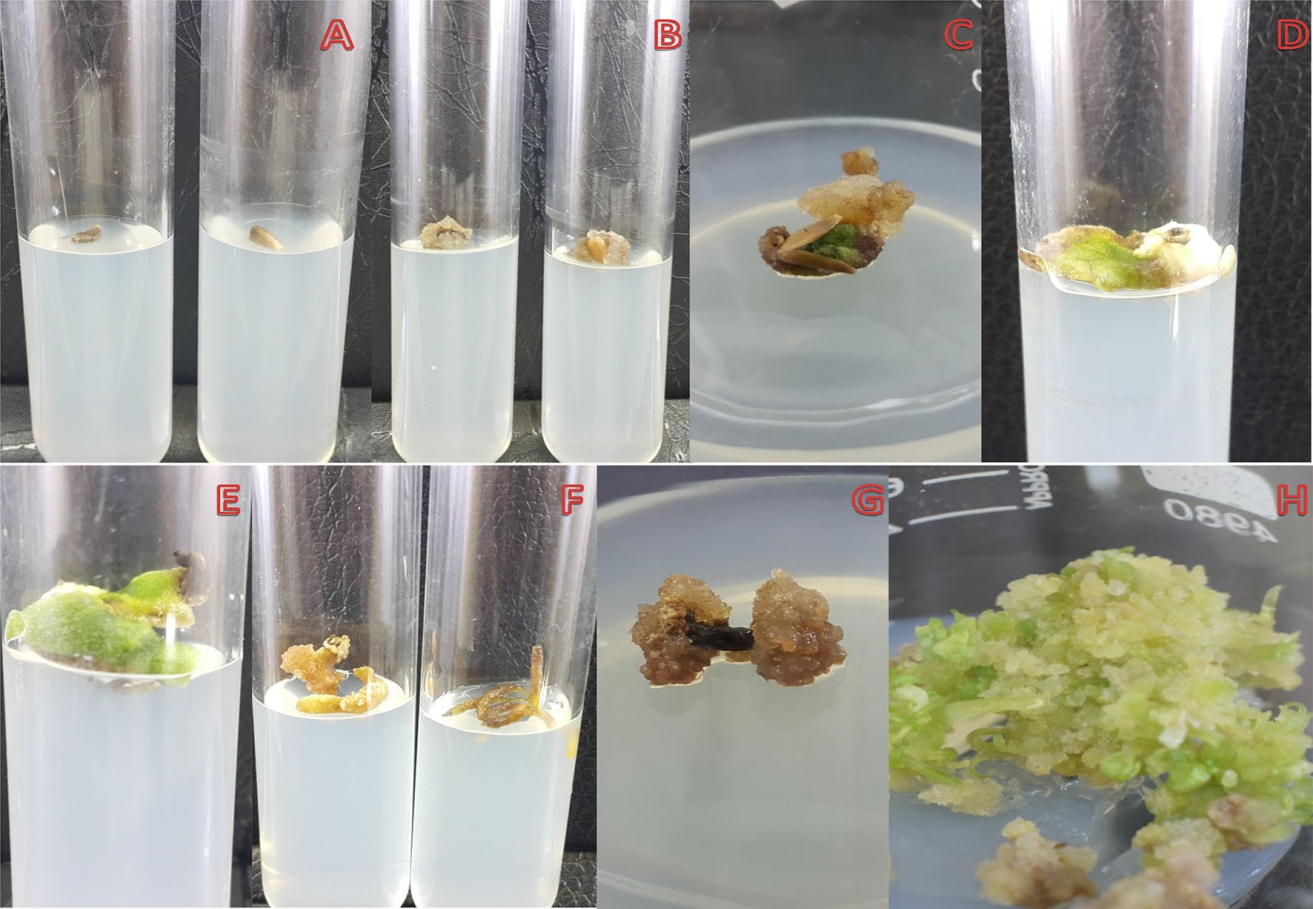
In-vitro callus induction of *S. costus* using seeds (**A**, **B**, **C**), leaf (**D**, **E**), roots (**F**) and nodes (**G**) as an explant.

Callus response was influenced by hormonal combinations as well as the type of explant used. The callus response varied *i.e*. 80–95%, 78–90%, 70–95% and 65–80% for seeds, leaf, petiole and root explants, respectively (Fig. 1A-H). Similarly, explants were grown on MS media alone (as control) for 14 days and no callus induction or regeneration was observed and therefore, these results are not included. Maximum amount of callus tissue per seed explant was formed on MS media agitated with 2,4-D (0.5 mgL^−1^) and Kinetin (1.0 mgL^−1^) as demonstrated in Fig. 1A-C. The colour of callus ranged from white to dark brown. Successful callus initiation was observed after 13, 15, 15 and 16 day of culturing from seed, leaf, petiole and root explants respectively (Table 2). It was also noted that subculture of callus into new media increased the callus biomass. Maximum callus growth from seed (1.86 g), leaf (1.65 g), petiole (1.42 g) and root (1.14 g) were record at 2, 4-D (0.5 mgL^−1^) and Kinetin (1.0 mgL^−1^) after twenty-eight days of culture (Table 2). Higher concentration of 2,4-D reduced callus induction and it was observed that the colour changed to brown with hard texture, followed by necrosis. Although 2,4-D is a synthetic plant growth regulator, its role in callus induction is highlighted for *S. costus*. Previous studies have also reported the efficacy of exogenous 2,4-D in other medicinal plants. Hassan et al. (2009)^20^ and Sen et al. (2014)^21^ have shown the positive role of 2,4-D plant growth hormones in culture media of *W. somnifera, I. obscura*, *A. precatorius* and *C. halicacabum* and their results are in agreement to those mentioned here. The effect of 2,4-D in combination with Kinetin demonstrated the potential of a synthetic plant growth regulators in the production of callus from seeds, leaf, petiole as well as root explants of *S. costus* as a potent plant growth regulator.

### Shoot bud initiation

**Figure 2:**
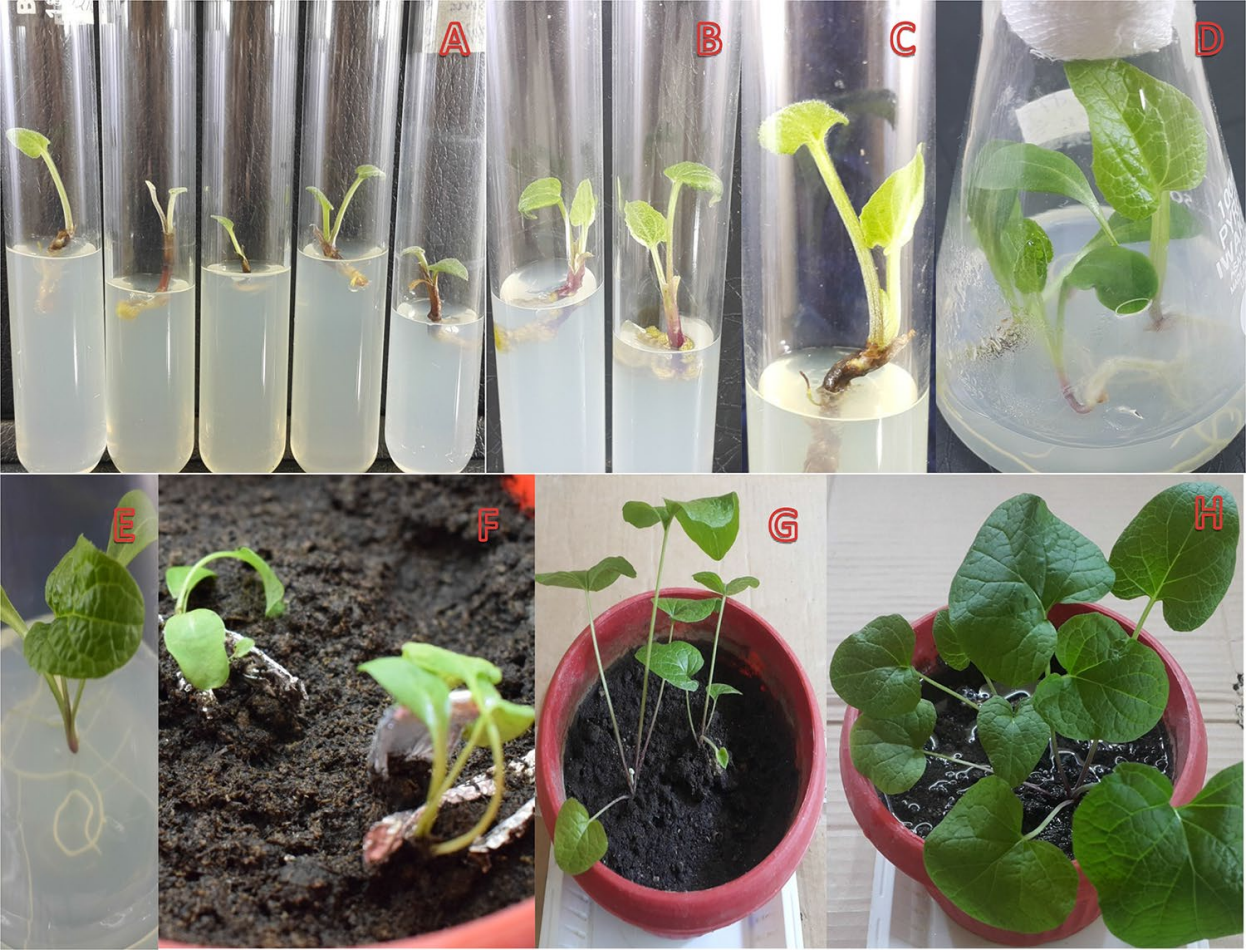
In-vitro Propagation of *S. costus* using nodal explant (**A**) Auxiliary buds, (**B**) Shoot buds initiation, (**C**) Multiple shoot initiation, (**D**, **E**, **F**) Mature plantlets, (**E**, **F**) Roots initiation, (**G**, **H**) Acclimatization of plants.

Auxiliary buds induction was observed after 15 to 20 days of culturing (Fig. 2A,B). The earliest shoot bud initiations were observed on media agitated with BAP (2 mgL^−1^), NAA (1 mgL^−1^) and GA3 (0.25 mgL^−1^). Higher concentration of BAP resulted in earlier buds induction. The analysis revealed BAP had a marked influence on the rate of induction. Similarly, BAP in low concentration, the induction rate was 64% and the lateral buds sprouted late. In addition, new buds were relatively thinner and delicate. ANOVA showed that shoot bud initiation was highly significant among the treatments (Table 3). Previous studies have also indicated that high level of BAP and low GA_3_ induced greater response to shoot buds initiation^22^. Similarly, BAP here was most effective for bud induction. GA_3_ contributes to the initiation and elongation of auxiliary buds and expansion of leaves^23^. Further, GA_3_ regulates the growth and development of plants, mainly by stimulating mitotic division and cell elongation^24^. It was found that high level of GA_3_ effectively increased stem length, while lower GA_3_ concentration inhibited potato shoot growth^25^. Further, GA_3_ has long been used to break dormancy and to stimulate shoot elongation in different species of magnolias^26^. In line with the previous reports, it was also observed that BAP in combination with GA_3_ was important for bud initiation, reducing time for buds initiation as well as resulted in stronger buds^27^.

**Table 3:** Influence of different plant growth regulators on buds initiation and Range analysis. Vigorous and green buds (+ + +); healthy buds (+ +); weak bud ( +). Each Value represents the mean ± SE of five replicates. Significant deference at P ≤ 0.05, x^-^ ± Sd- average ± Standard deviation, x ± SE – average ± Standard error.

Treatment	BAP mgL^-1^)	NAA mgL^-1^	GA_3_ mgL^-1^	Bud induction rate (x- ± SE)	Time to bud initiation	Growth state of bud
T7	0.5	0	0	19.40 ± 1.50^A^	19 Days	+
T8	0.5	0.25	0	17.80 ± 1.02^AB^	18 Days	+ +
T9	1.0	0	0.25	17.60 ± 0.37^AB^	18 Days	+ + +
T10	1.0	0.25	0.25	17.00 ± 0.44^AB^	17 Days	+ + +
T11	2.0	1.0	0.25	15.60 ± 0.87^B^	15 Days	+ + +
T12	2.0	1.0	1.0	16.40 ± 0.67^B^	16 Days	+ + +

### Shoot bud proliferation

Full strength media augmented with BAP (0.1 mgL^−1^), NAA (0.25 mgL^−1^) and Kinetin (0.25 mgL^−1^) proved best for shoot bud proliferation and elongation (Fig. 2C). Significant differences were observed in multiplication rate and numbers of shoots between T7, T8 and T9, although T13 is significantly different from T14 and T15, while T13 and T14 are not significantly different (Table 4). Das et al. (2020)^28^ has also recorded maximum number of shoot/explant of *B. polystachyon* with combination of BAP (13.32 μM) and NAA (0.53 μM). Several medicinal plants such as, *C. paniculatus* ^29^ and *C. blumei* ^30^, have shown similar results and BAP with NAA have been reported as being the most effective in direct organogenesis. Our results are in alignment to those of Kaur et al. (1998)^31^, where 8–10 shoot/explants of *A. catechu* from nodal segment on media containing BAP (4.0 mgL^−1^) with NAA (0.5 mgL^−1^) were reported.

**Table 4:** The influence of different concentrations of plant growth regulators on bud proliferation of *S. costus*. vigorous and green buds (+ + +); healthy buds (+ +); weak buds ( +). Each value represents the mean ± SE of five replicates.

Treatments	BAP (mgL^-1^)	NAA (mgL^-1^)	Kinetin (mgL^-1^)	shoots numbers per plant (≧0.5 cm) (mean ± SE)	shoots numbers per plant (≧0.5 cm) (mean ± SE)	Total shoot length (≧0.5 cm) (mean ± SE)
T13	0.5	0.25	0	2.20 ± 0.44	2.20 ± 0.20^B^	2.19 ± 0.26^B^
T14	1.0	0.25	0.25	4.00 ± 0.70	4.00 ± 0.31^A^	2.50 ± 0.30^AB^
T15	1.5	0.25	0.5	2.80 ± 0.83	2.80 ± 0.37^B^	3.11 ± 0.33^A^

### Total shoot length

Average shoot length ranged from 2.19 to 3.11 cm among the treatments. Maximum shoot length was recorded for media fortified with BAP (1.5 mgL^−1^), NAA (0.25 mgL^−1^) and Kinetin (0.5 mgL^−1^). While, the minimum shoot length was recorded in media with BAP (0.5 mgL^−1^), NAA (0.25 mgL^−1^) without Kinetin (Fig. 2D,E). ANOVA revealed significant variation in T15 compared to T13 and T14 (Table 4). The addition of even smaller amounts of BAP or NAA help inducing adventitious shoot formation by increasing propagation coefficient^32^. Other researchers have also reported that highest shoot length (3.73 ± 0.14 cm) of *S. rebaudiana* was observed on MS media supplemented with BAP (2.0 mgL^−1^) and IAA (0.25 mgL^−1^) after 15 days of culturing^33^. Additionally, higher concentrations of BAP reduces shoot length, which is in agreement to the known literature^34^.

### Root initiation, number of roots and total root length

Roots induction is a critical step in successful in-vitro propagation experiments; here combination of IAA (0.5 mgL^−1^) with IBA (0.5 mgL^−1^) resulted in earliest roots initiation (13 days), while IAA (0.1 mgL^−1^) delayed late root formation (19 days) (Fig. 2D-E). Further, IAA (0.5 mgL^−1^) in combination with IBA (0.5 mgL^−1^) resulted in earliest as well as plenty of lateral roots formation (Table. 5). ANOVA showed that TI6 was significantly different, while T17 and T18 had no significant variation (Table 5). IBA is a highly stable and potential auxin for roots induction^35^. Maximum numbers of roots (6.76) were recorded on full strength media supplemented with BAP (0.5 mgL^−1^), IAA (0.5 mgL^−1^) and IBA (0.5 mgL^−1^) (Fig. 2E). On the contrary, least number of roots per plant (3.84) were formed on media supplemented with IAA (1 mgL^−1^). Statistical analysis revealed that T16 and T18 varied significantly (Table 5). The in-vitro derived shoots on MS medium were supplemented with a range of concentrations of two auxins (IAA and IBA) for 75 days, it was observed that the lower concentrations of BAP (0.5 mgL^−1^) in combination with IBA (1 mgL^−1^) resulted in a higher root length (2.53 cm), while IAA (1mgL^−1^) and IBA (1 mgL^−1^) alone induced roots length of (1.5 cm) and (2.27 cm) respectively. Results showed that IAA in comparison to IBA reduced roots length when compared at the same concentration (Table 4). Statistical analysis showed that root length at T16 was significantly different from T17 and T18. Cheepala et al. (2004)^36^ reported that IAA is a widely used auxin for rooting in *A. stenosperma* and *A. villosa*. In several other plants species the promoting effect of IBA in rooting has also been reported^37^. In contrast, induction of rooting of *G. scabra* was obtained on NAA (0.3 mgL^−1^) and IAA (0.1 mgL^−1^) containing media^38^. Similarly higher percentage of rooting were obtained in half strength MS media with NAA (1.0 mgL^−1^), were as full strength medium with NAA (1.5 mgL^−1^) was the best media for rooting^10^. Bekheet (2013)^39^ has indicated that addition of IAA, IBA or NAA (1 mgL^−1^) induced rooting of in-vitro grown *P. dactylifera*. However, in the present study, IAA in combination with IBA was found to be the most efficient in multiple shoots induction, followed by IBA alone.

**Table 5:** The effect of different concentrations of IAA and IBA along with BAP on roots initiation, number of roots and total root length of *S. costus*. Each value represents mean ± SE of five replicates.

Treatment	BAP (mgL^-1^)	IAA (mgL^-1^)	IBA (mgL^-1^)	Days to root initation (mean ± SE)	Number of roots (mean ± SE)	Total root length (cm) (mean ± SE)
T16	0.5	1.0	0	18.80 ± 1.01^A^	3.84 ± 0.508^B^	1.50 ± 0.19^B^
T17	0.5	0	1.0	16.20 ± 0.80^AB^	5.32 ± 0.531^AB^	2.53 ± 0.21^A^
T18	0.5	0.5	0.5	13.80 ± 0.91^B^	6.76 ± 0.733^A^	2.27 ± 0.24^A^

### Acclimatization

The ultimate success of all in-vitro micro propagation endeavors heavily relies on the higher survival rates of such plantlets. Direct field transfers of the plantlets do not allow acclimatization of the in-vitro generated plants as they fail to establish successful interactions with the soil microbes and/or to sustain the environmental conditions^40^. Here, well rooted micro propagated plantlets were transferred into plastic pots containing autoclaved garden soil, farmyard soil, and sand (2:1:1) as shown in Fig. 2F–H. The plants were then acclimatized in the growth room at 27 °C temperature for 2 weeks followed by another 3 weeks at room temperature under laboratory conditions. Finally, 35–40 days old plantlets were transferred to nursery where, morphological anatomical and growth characteristics were observed (results not shown) and survival efficiency recorded. Out of 92 plantlets, 80 (87%) could successfully acclimatize and the relatively low mortality rate here is likely to be due to the biohardening of the micropropagated plants achieved prior to their nursery transfer. Similarly, we have given water to the plantlets after 6 days interval and that too very close to the roots and have avoided leaves. This approach has been previously reported beneficial for in-vitro raised plants^41^ and the survival rate could be raised significantly higher if biotization of the explants is attempted^42^.

### Phytochemical variation

#### Total sugar contents

**Figure 3:**
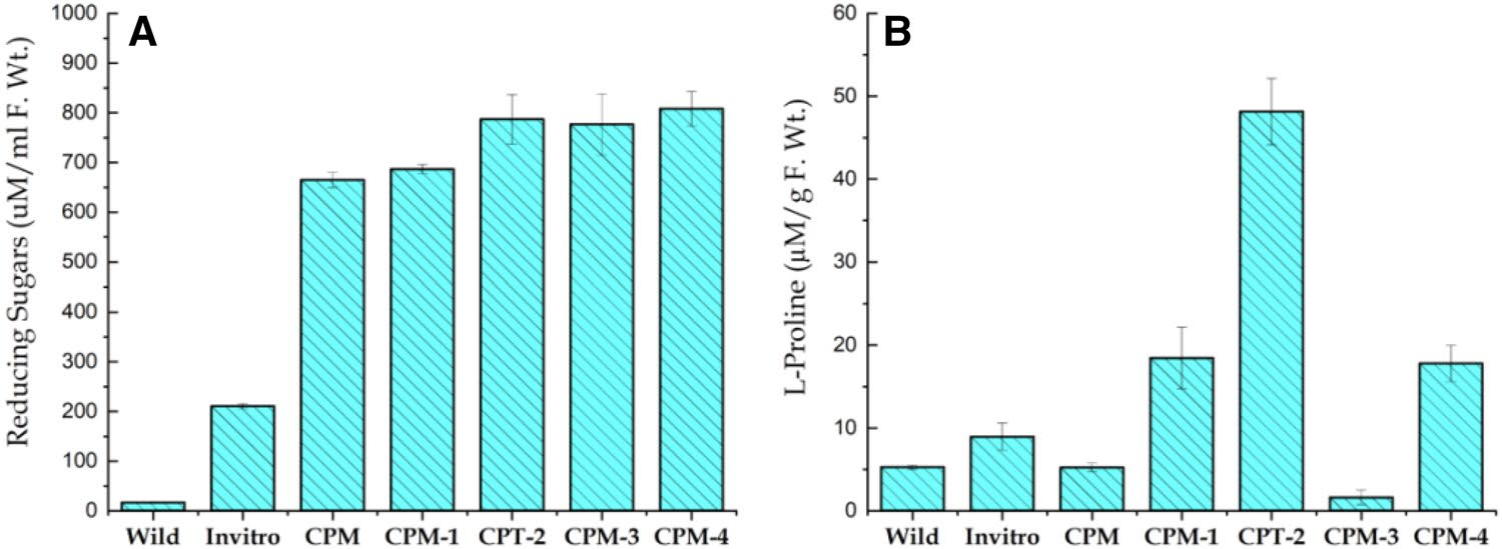
Variation in the Total Sugar contents (**A**) and proline contents (**B**) of *S. costus* collected from wild, in-vitro propagated plant and induced callus. Different stress for phytochemical comparison of callus grown on simple callus promoting media (CPM) supplemented with Kinetin (0.5 mgL^-1^) and 2.4-D (1.0 mgL^-1^) and callus subjected to various stresses i.e. 60 gL^-1^ D-Sorbitol stress (CPM-1), 60 gL^-1^ D-Manitol (CPM-2), 5 gL^-1^ Poly ethylene glycol 600 (CPM-3) and 60 gL^-1^ Sucrose (CPM-4).

Total sugar contents revealed significant variation with treatments. Maximum sugar contents (808.326 µM/ml) was observed in callus cultured on CPM-4 supplemented with 60 gL^−1^ sucrose, while the lowest sugar contents (16.64 µM/ml) was noted in wild plants (Fig. 3A). Accumulation of sugars contents in different parts of plants increases in response to a variety of environmental stresses^43^. The accumulation of total sugars is associated with adaptation of plants to various environmental stresses^44^. The results shown here are in agreement to earlier findings where salinity increased total sugar contents in leaves of in-vitro propagated *P. euphratica*. Similarly, addition of NaCl (250 mmoll^−1^) increased sugars contents by 2.7 times^45^. In calli of *M. arborea* total sugars account for about 90% of the total dry weight and there were no significant differences. The remarkable differences between the embryogenic and non-embryogenic calli of *M. arborea*, was the amount of sugar found in embryogenic calli^46^. A similar trend with total sugars accumulation was also detected in *P. kurroa*^47^. In line results are also shown for the total sugars in selected calli of *D. caryophyllus* subjected to different concentrations of culture filtrate that were significantly higher than those of non selected calli^48^.

#### Proline content

Since, callus promoting media was used as a control; the stresses imposed increased proline content in callus from 1.63 to 48.14 mg/g F.Wt. The variability in proline content among the different treatments were highly significant as shown in Fig. 3B. Maximum proline contents was noticed in CPM-2 agitated with 60 gL^−1^ D-Manitol (48.14 mg/g) whereas, minimum was observes in CPM-3 supplemented with 5 gL^−1^ Poly ethylene glycol 600 (1.63 mg/g). In brief, different stresses enhanced proline contents in *S. costus* callus as follows: callus treated with 60 gL^−1^ D-Manitol (48.14 µM/g) > 60 gL^−1^ D-Sorbitol (18.45 µM/g) > 60 gL^−1^ Sucrose (17.79 µM/g) > 5 gL^−1^ Poly ethylene glycol 600 (1.63 µM/g). Results presented here are in general agreement to earlier reports where, authors have reported proline accumulation in calli of sugarcane grown on different concentration of PEG^49^. Similarly, total proline level of 20% PEG selected calli was reported to be 17 times higher than the non-selected calli of *O. sativum*^50^. Pradhan et al. (2021)^51^ reported on the increasing trends in proline contents (0.798 µMg^−1^) in mango callus subjected to 15% PEG stress as compare to control (no PEG) with the value of 0.080 µM g^−1^ FW. Similar increase in proline contents is also mentioned for *H. annuus*^52^ as well as rice in response to PEG stress^53^. Previously, D-sorbitol stress has resulted in more than four-fold increase in proline level in maize seedling^54^ and these results are in full agreement to those reported here.

#### Total flavonoids

**Figure 4:**
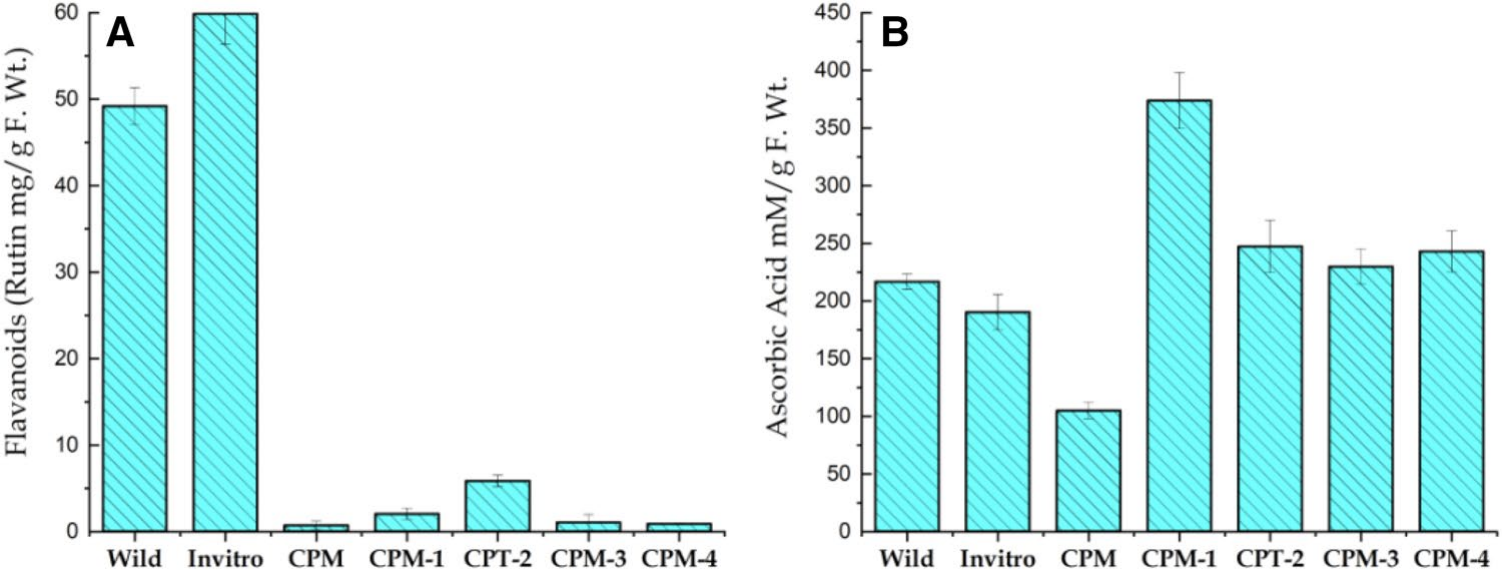
Variation in the Total Flavenoids (**A**) and Ascorbic acid (**B**) contents of *S. costus* collected from wild, micro propagated plant and induced callus grown on simple callus promoting media (CPM) supplemented with Kinetin (0.5 mgL^-1^) and 2.4-D (1.0 mgL^-1^) and callus subjected to various stresses i.e. 60 gL^-1^ D-Sorbitol stress (CPM-1), 60 gL^-1^ D-Manitol (CPM-2), 5 gL^-1^ Poly ethylene glycol 600 (CPM-3) and 60 gL^-1^ Sucrose (CPM-4).

Favonoids have protective functions for plants growing in soils that are rich in toxic metals. Here flavonoids contents showed significant variation among wild and in-vitro propagated plant as well as induced callus of *S. costus*. This variation in flavonoids content ranged from 0.90 to 59.89 mg/g. Results showed that in-vitro propagated plants had the highest flavonoids contents (59.892 mg/g) followed by plants collected from wild (49.199 mg/g). Similarly, lowest flavonoids contents were reported in callus agitated on CPM-4 supplemented with 60 gL^−1^ Sucrose (Fig. 4A). Comparing wild and in-vitro propagated plants, callus contained relatively scarce amount of flavonoids, and this is most likely due to plants were grown in laboratory environment with very much uniform environmental conditions. In natural habitats plants are well adapted and have evolved mechanisms to minimize injuries under extreme environmental conditions. The accumulation of flavonoids in the cells resulted by osmotic stress are often associated with a mechanisms that allow plants to tolerate harmful effects of water shortage. Further, accumulation of these solutes lower the osmotic potential of plant tissues at cellular level and hence allowing plants to sustain growth in stressful environments^55^. Ibrahim et al. (2018)^56^ have also noted maximum level of total flavonoids in wild or natural *P. barbatus* as compared to in-vitro propagated plants and callus^57^.

#### Ascorbic acid contents

Maximum accumulation of Ascorbic acid (373.801 mM/g) was recorded in callus cultured on CPM-1 supplemented with 60 gL^−1^ D-Sorbitol, followed by CPM-2 that had subjected to (60 gL^−1^) D-Manitol stress (373.801 mM/g), while minimum amount was observed in callus grown on CPM (104.95 mM/g) (see Fig. 4b). Kamal et al. (2020)^58^ have studied the optimization of suitable media for callus induction and ascorbic acids accumulation in Chinese cabbage cultivars. The authors have found maximum ascorbic acid accumulation in callus of root explant cultured on TDZ (1.0 mgL^−1^), NAA (0.25 mgL^−1^) and AgNO3 (5.0 mgL^−1^), while minimum ascorbic acid was noted for callus grown from hypocotyl tissues cultured on TDZ (1.0 mgL^−1^), NAA (1.0 mgL^−1^) and AgNO3 (9.0 mgL^−1^). Likewise, using the DPPH assay, free radical scavenging and antioxidant potential of in-vitro propagated *S. corymbosa* plants were compared with the wild plants in where the wild plants have shown highest free radical scavenging activity compared to the in-vitro propagated plants^59^.

#### Total phenolic compounds

**Figure 5:**
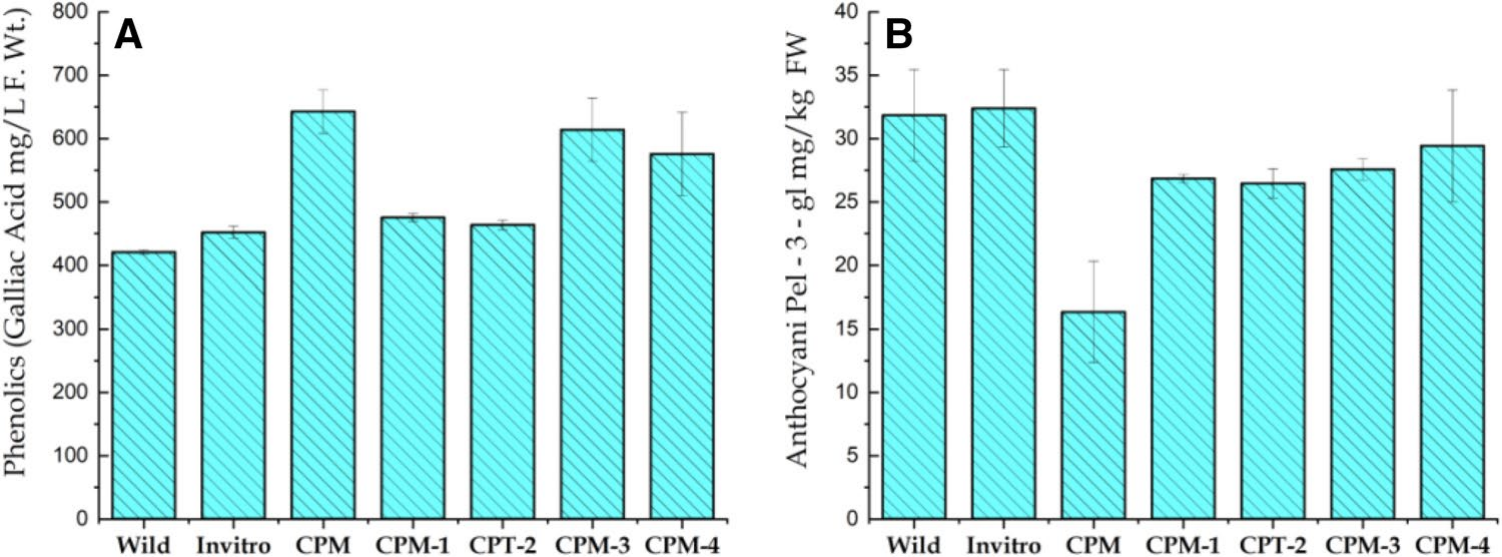
Variation in the Total Phenolics (**A**) and Anthocyanin (**B**) of *S. costus* collected from wild, micro propagated plant and induced callus. Different stresses for phytochemical comparison of callus grown on simple callus promoting media (CPM) supplemented with Kinetin (0.5 mgL^-1^) and 2.4-D (1.0 mgL^-1^) and callus subjected to various stresses i.e. 60 gL^-1^ D-Sorbitol stress (CPM-1), 60 gL^-1^ D-Manitol (CPM-2), 5 gL^-1^ Poly ethylene glycol 600 (CPM-3) and 60 gL^-1^ Sucrose (CPM-4).

Phenolics compounds represent a diverse array of plant secondary metabolites, which are predominantly used as powerful scavengers of free radicals (Pietta, 2000)^60^. Here, highest phenolic contents (642.72 mgL^−1^) accumulated in calli cultured on CPM when compared to wild or in-vitro propagated plantlet. Similarly, lowest levels of phenolic compounds (420 mgL^−1^) were recorded in plants collected from wild (Fig. 5A). Increase in phenolic compounds accumulation (37% and 34%) was observed in callus treated with 100 mgL^−1^ yeast extract and 50 mgL^−1^ salicylic acid^24^. These finding are supported by those given in El-Nabarawy et al. (2015)^61^, where the culture medium supplemented with low concentration of yeast extract increased phenolic accumulation in micro propagated plants. Furthermore, Gorni and Pacheco (2016)^62^ have reported that *A. millefolium* treated with 0.5 and 1.0 mM salicylic acid significantly increases phenolic contents. A slight increase in total phenolic content was found in callus treated with glycine (200 mgL^−1^), yeast extracts (500 mgL^−1^) and salicylic acid (100 mgL^−1^). This increase of phenolic contents in callus cultures was related to mitochondrial activity; that is, while the cell dehydrogenase activity (FADH2/NADH) and the cytochrome C-oxidase decrease, the production of phenolic compounds increases^63^. On the other hand, variation in total phenolics within the mother source plant, micropropagated plants and callus subjected to different stresses may be attributed to changes in the levels of various phytohormones or other endogenous physiological pathways that occur in plant^64^. Also synthetic plant growth regulators used during the micro propagation pathways make a significant contribution in the production of secondary metabolites within the in-vitro cultured cells and tissues by controlling the expression of genes involved in the synthesis of secondary metabolites such as shikimate and flavonoids^65^.

#### Total anthocyanin

Anthocyanin contents were detected in the form of Pelargonidin-3-glucoside per kilogram of fresh sample. In the current analyses, in-vitro propagated plant possessed highest amounts of anthocyanin (32.39 mg/kg) followed by wild (31.84 mg/kg) whereas, lowest amount of anthocyanin was recorded in callus grown on CPM (with no stress see Fig. 5B). A similar trend in callus cultures of *A. cordata* anthocyanin accumulations was achieved with a combination of either NAA or 2,4-D and Kinetin in comparison with auxins or cytokinins^66^. Similarly, maximum total anthocyanin contents (3.3 to 7.4 CV/g) was reported from cultures on moderate level (40 and 50 mM) of total nitrogen^67^. Our results are in agreements with several authors where, highest values of all estimated anthocyanin were recorded in shootlets of *A. leptopus* obtained from MS supplemented with 2iP (0.4 mg/l) and IBA (0.1 mgL^−1^)^68^. Similarly, calli derived from style of *Crocus sativus* showed anthocyanin pigment of 3.75 × 10^–7^ mg g^−1^ on media supplemented with NAA (2 mgL^−1^) and TDZ (1 mgL^−1^) compared to that of calli produced from corm 2.52 × 10^–7^ mg g^−1^ DW^69^.

#### GC–MS analysis of callus ethyl acetate extract

**Figure 6:**
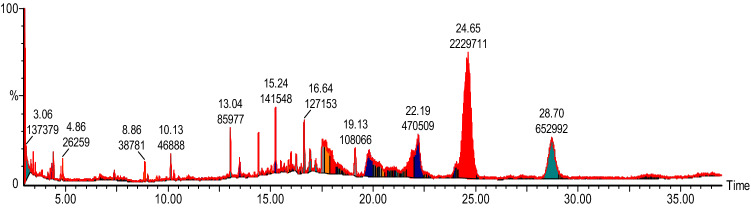
GC–MS Chromatogram of ethyl acetate extracts of callus grown on simple callus promoting media (CPM) supplemented with Kinetin (0.5 mgL^-1^) and 2.4-D (1.0 mgL^-1^).

**Figure 7:**
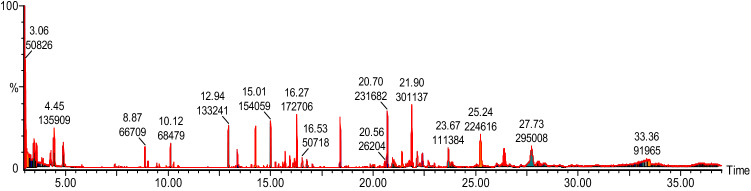
GC–MS Chromatogram of ethyl acetate extracts of callus subjected to 60 gL^-1^ D-Sorbitol stress (CPM-1).

GC–MS chromatograms of ethyl acetate extract of callus and callus subjected to different stresses revealed the presence of 24 compounds. The active principles with their retention time, molecular weight and peak area (%) of the identified compounds that could contribute the medicinal quality of the plant are summarized in Table 6. The major components in the CPM extract were Propanic acid, 2-methyl-,3,7-dimethyl-2,6-octadienyl ester and Selina-3,7(11)-diene. The analysis of GC–MS chromatogram showed peaks of various phytochemical constituents present in ethyl acetate CPM extracts (Fig. 6). In contrast, major components identified in CPM-1 were Nonadecane, 2,6,10,14,18-pentamethyl, Nonadecane,2,6,10,14–tetramethyl and 6-tetradecane sulfonic acid, butyl ester (see Table 6, Fig. 7). In CPM-2, major phytocomponents were Octacosane,1-Iodo, Octadecane-2,6,10,14-tetramethyl, Nonadecane, 2,6,10,14,18-pentamethyl and Nonadecane ,2,6,10,14–tetramethyl. Similarly, in CPM-3 and CPM-4 major phytocomponents recorded were Nonadecane,2,6,10,14–tetramethyl, Eicosane,2,6,10,14,18-pentamethyl, Tetrapentacotane and Nonadecane,2,6,10,14,18-pentamethyl, Nonadecane, 2,6,10,14–tetramethyl and Heneisane respectively (see supplementary Figs. 8–10 and Table 6). While, the phytocomponents such as Octadecane-2,6,10,14-tetramethyl and Hentriacontane were present in all the tasted samples. High amount of Octadecane-2,6,10,14-tetramethyl was observed in CPM-2, while the amount of Hentriacontane was higher in CPM-1. Previously, Gwari et al. (2013)^3^ has reported 41 aromatic compounds from essential oil of *S. costus* roots extracts. Among the identified compounds Aldehyde like (7Z, 10Z, 13Z)-7, 10, 13-hexadecaterinal, ketones like dehydrocostus lactone, alcohols like elemol, g-costol, vulgarol B, valerenol, and terpinen-4-ol, etc. were found a major component. In addition, Esters and acids were found to be completely absent in root extracts of *S. costus*. Srinivasan et al. (2016)^70^ studied the chemical compounds in costus oil and observed that n-hexadecanoic acid to be the major constituent in all examined essential oil accompanied with other fatty acids, hydrocarbons and mono-,di- sesquiterpenes. Recently, Deabes et al. (2021)^71^ identified 14 components from *S. costus* ethyl acetate extracts. Compounds like Butanedioic acid and 2TMS derivative were recorded in highest percentage followed by D-(-)-Fructofuranose, pentakis (trimethylsilyl) ether (isomer1), Androstan-17-one, 3-ethyl-3-hydroxy-, (5.alpha.)-, Caffeic acid, 3TMS derivative and L-(-)- Sorbofuranose and pentakis (trimethylsilyl) ether. This great variation in phytocomponents of *S. costus* may be attributed to factors related to ecotype, chemotype, phenophases and the variations in environmental conditions such as temperature, relative humidity, irradiance and photoperiod. Moreover, the genetic background may also affect the chemistry of secondary metabolites of plants^72^. Furthermore, exposure to various type stresses may result in drastic epigenetic modifications thereby, changing the transcriptional activities and the overall transcriptomic profile^73^. Recently it has been shown that stable phenotypes can be generated through epigenetic modifications and thereby increasing the success and survival of plants in their natural habitats. Although, we have not studied any such epigenetic modifications here, but these are very likely targets and are important consideration to be included in future studies.

**Table 6:** Phytocomponents identified in ethyl acetate extract of *S. costus* callus grown on simple callus promoting media (Kinetin: 0.5mgL^-1^ and 2.4-D: 1.0 mgL^-1^) and callus subjected to various stresses i.e. 60gL^-1^ D-Sorbitol stress (CPM-1), 60gL^-1^ D-Manitol (CPM-2), 5gL^-1^ Poly ethylene glycol 600 (CPM-3) and 60gL^-1^ Sucrose (CPM-4).

RT	Compound name	Molecular mass	Cas number	CPM	CPM-1	CPM-2	CPM-3	CPM-4
				% area	% area	% area	% area	% area
10.13	Dodecane,2,6,10-trimethyl	212	3891–98-3	1.35	3.045		1.97	
13.04	Hexadecane	226	544–76-3	2.48	5.92		4.57	3.73
14.23	Bezene,1,4-bis(1,1-dimethylethyl)	190	1012–72-2			8.67	5.31	5.17
14.41	Benzene, 1,3bis(dimethylethyl)	190	1014–60-4	2.26				
14.92	Nonadecane,2,6,10,14 tetramethyl	324	55,124–80-6				7.55	4.31
14.98	Octacosane,1-Iodo	520	900,406–32-2			12.34		
15.24	Octadecane-2,6,10,14-tetramethyl	310	54,964–82-8	4.08	6.85	12.34	3.20	9.40
16.64	Hentriacontane	436	630–04-6	3.66	7.68	7.47	1.89	3.87
18.4	Heptadecane,2,6,10,15-Tetramethyl	296	54,833–54-6		7.90			5.38
18.31	Tritetracontane	604	7098–21-7				5.46	
18.32	Dodecane,1-fluoro	188	334–68-9			9.89		
19.13	Carbonic acid,decyl undecyl ester	356	900,383–16-0	3.11				
20.7	6-tetradecanesulfonicacid,butyl ester	334	20,028,280–27-4		10.30			10.62
21.6	Nonadecane,2,6,10,14,18-pentamethyl	338	55,191–61-2		13.39	12.76	10.47	18.60
21.7	Nonadecane,2,6,10,14 -tetramethyl	324	55,124–80-6			22.43	16.90	14.28
21.9	Eicosane,2,6,10,14,18-pentamethyl	352	51,794–16-2		13.39		16.01	
23.67	Heneisane	296	629–94-7		4.28	7.04		12.89
24.65	Propanic acid,2-methyl-,3,7-dimethyl-2,6-octadienyl ester	224	2345–24-6	64.24				
25.24	Alpha-maaliene	204	489–28-1		9.31			
25.59	Dotriacontane	450	544,854				12.29	
26.4	Tetrapentacotane	758	5856–66-6		7.795		14.37	5.92
27.52	Dotricotane	450	544,854					5.84
27.73	Triacotane	422	638–68-6		10.11	7.04		
28.7	Selina-3,7(11)-diene	204	6813–21-4	18.81				
Total identification	100	99.99	100	100	100

## Conclusion

Efficient protocols for large scale callus induction of four explants (seeds, leaf, petiole and internodes) as well as micro propagation from auxiliary buds of *S. costus* were developed. Callus formation was greatly influenced by type of explant used and maximum callus tissue with minimum time taken was record for seed explants. The best response to direct organogenesis was observed on media fortified with BAP (2.0 mgL^−1^), NAA (1.0 mgL^−1^) and GA3 (0.25 mgL^−1^). Micropropagated plantlets suffer high mortality due to their slow acclimatization to *ex-vitro* conditions. In spite of the prior limited success with Asteraceae members in inducing roots during tissue culture and acclimatization; here, the regenerated plantlets had 87% of survival rate. We argue this survival rate could be further improved through biotization of micro propagated plants with endophytic bacteria and fungi. Here, phytochemical characterization and variability in metabolites such as total sugars, proline, flavonoids, ascorbic acid, phenolics and anthocyanin is recorded from callus, wild as well as micro propagated plantlets. It is also demonstrated that *S. costus* callus is rich source of various bioactive compounds as indicated in the GC–MS profiles. The remarkable variation in the secondary metabolites may be partly explained by the preexisting genetic variation within the populations of *S. costus,* understanding the role of epigenetic regulation in response to environmental stimuli, particularly in response to stresses is of paramount significance to the stability and survival of the plants in their natural habitat. The current work on this critically endangered species provides a baseline for future work including application of the newly evolved biotechnological tools that may speed up and ensure sustainability of the plant species, thereby enhancing conservation and management of *S. costus* in natural ecosystems.

## Approval and compliance with regulation

The study was formally authorized by the Directorate of Academics and Research Hazara University Mansehra, Pakistan. Experimental research and field studies on selected plant, including the collection of plant material comply with relevant institutional guidelines and legislation.

## Statement for submission of specimen to University herbarium

Specimen was collected from Makra, mountain peak (alt 3,878 m), situated in District Mansehra, KP Pakistan. Date 10–07-2019, GPS (lat 34.57439º N, long 073.49580º E), collected by Ajmal Khan, Azhar Hussain Shah, and Abdul Majid, 57 (HUP).

## Supplementary Information

Supplementary Information.

## Data Availability

All data are available in the manuscript.
