# Supplementary material for: In-vitro propagation and phytochemical profiling of a highly medicinal and endemic plant species of the Himalayan region (Saussurea costus)
Source: Sci Rep. 2021 Dec 8;11:23575. doi: 10.1038/s41598-021-03032-1 (PMC8654858; doi:10.1038/s41598-021-03032-1)
Supplement: Supplementary file 1 — Supplementary Information. [file 41598_2021_3032_MOESM1_ESM.doc]

**Fig. 8** GC-MS Chromatogram of ethyl acetate extracts of callus subjected to 60gL^-1^ D-Manitol stress (CPM-2).

**Fig. 9** GC-MS Chromatogram of ethyl acetate extracts of callus subjected to 5gL^-1^ Poly ethylene glycol 600 stress (CPM-3).

**Fig. 10** GC-MS Chromatogram of ethyl acetate extracts of callus subjected to 60gL^-1^ Sucrose stress (CPM-4).
